# Surgical success following robotic upper urinary tract reconstruction, results from a global network of healthcare organizations

**DOI:** 10.1007/s11701-025-02951-9

**Published:** 2025-11-08

**Authors:** Zachary J. Prebay, Adam Schneider, Sohan Shah, Mauro Dispagna, Mihir S. Shah

**Affiliations:** 1 Department of Urology, Sidney Kimmel Medical College at Thomas Jefferson University, Philadelphia, USA; 2 Department of Urology, Sidney Kimmel Medical College, Thomas Jefferson University, 1025 Walnut St. Ste. 1100, Philadelphia, PA 19107 USA

**Keywords:** Robotics, Pyeloplasty, Ureteral reimplantation, Ureteroplasty

## Abstract

**Introduction:**

Available data on robotic ureteral reconstruction procedures is concentrated from high volume centers. To better understand generalizability of success, we used a network of healthcare organizations (HCOs) to evaluate surgical success following robotic-assisted pyeloplasty (RP), ureteral reimplantation (RR), and ureteroureterostomy/ureteroplasty (RU).

**Methods:**

We searched the TriNetX database for adult (>18 years old) patients undergoing RP, RR and RU. Our primary outcome was need for drain placement (nephrostomy or ureteral stent) from 4 weeks to 10 years postoperatively. We evaluated risk factors (RF) for drain placement (vascular disease, kidney disease, diabetes (DM), smoking history, radiation history, age, body mass index (BMI, kg/m^2)) using hazard ratios, with significance when 95% confidence interval does not include 1.0.

**Results:**

Analyses were run in October 2024. There were 1,324 RP patients from 30 HCOs, 464 RR patients from 22 HCOs, and 875 RU patients from 27 HCOs. Respectively, 10.8%, 11.7%, and 7.9% of patients needed a drain. DM (HR 2.0 (1.04–3.68)), smoking (HR 2.2 (1.34–3.49)), and BMI > 25 (HR 1.6 (1.07–2.34)) were RFs for drain placement following RP. DM (HR 2.8 (1.18–6.63)) was also a RF for RR patients. Radiation was a RF for both RR (HR 2.9 (1.03–8.09)) and RU (HR 3.7 (1.006–13.9)) patients. Age > 50 years (HR 2.3 (1.16–4.49)) was a RF for RU patients.

**Conclusions:**

We report outcomes of robotic ureteral reconstruction from a variety of HCOs. This data helps describe the real world experience of patients undergoing robotic upper urinary tract reconstruction.

## Introduction

Ensuring proper urinary drainage is a fundamental tenet of Urology. Obstruction at any level from the kidney to the urethra can result in significant morbidity and even mortality. Ureteral stents or percutaneous nephrostomy tubes can be used to bypass obstruction at the level of the ureteropelvic junction, ureterovesical junction, or the ureter itself [1]. However, these drains require chronic changes and may cause adverse side effects, which is burdensome on patients and providers and may have a substantial negative impact on quality of life [2, 3]. Thus, upper tract urinary reconstructive surgeries were developed, first with open and later with minimally invasive approaches [4, 5]. In modern times, many of these cases are done with the Da Vinci robot (Intuitive Surgical, Sunnyvale, CA), and the data suggests that these surgeries are highly successful [6]. The robotic platform offers many benefits, such as improved access to the pelvis and retroperitoneum, precision in fine tissue handling, the use of visualization aids such as indocyanine green to assess tissue perfusion, and increased magnification [7, 8]. 

However, many of these procedures are relatively uncommon. This is especially true for ureteroureterostomy/ureteroplasty and ureteral reimplantation, with much of the data being retrospective and either single-institutional or small multi-institutional studies [6]. Further, most of the data on success rates comes from a few, high-volume centers with well-known experts in the field. Therefore, it is reasonable to question whether their success rates can be generalized to all patients undergoing these procedures with other providers or institutions.

In this context, our objective was to utilize the power of a large, multi-institutional research dataset to aggregate real world outcomes data following multiple robotic assisted ureteral reconstruction procedures. Additionally, we sought to analyze various risk factors that could result in poor outcomes. By comparing data from this larger consortium of healthcare organizations (HCOs), we can better counsel our patients on their likelihood of success, outside of the published data from centers of excellence. We hypothesize that patients in our larger analysis will still experience good success rates following these procedures. If so, this may reassure other urologists interested in offering robotic reconstruction and also improve access to care for more patients.

## Methods

### Data source

We accessed the TriNetX Research Network (www.trinetx.com), which is a multi-national collaborative research enterprise containing real-time data from the electronic health records of over 100 million patients located in 107 HCOs at the time of analysis. The database continually updates but only reports data coded into a patient’s EHR from up to 20 years prior to the date of analysis (2004–2024), excluding those undergoing the index event prior to this date. The dataset includes data on demographics, medical diagnoses, procedures, lab values, and medications. Documentation in TriNetX was reliant on a combination of International Classification of Diseases (ICD) and Current Procedural Terminology (CPT) coding. TriNetX maps ICD-9 and ICD-10 codes to the ICD-10-Clinical-Modification extension. The data within TriNetX reflects how the information is received from the HCOs, and like all registry studies, degrees of assumptions must be made regarding the quality, reliability, and accuracy of this, and any, large, de-identified data set. Once HCO data are transformed into the TriNetX proprietary data schema the data undergoes extensive data quality assessment that includes rejection of records that do not meet their quality standards. Due to the de-identified nature of this dataset, our study was considered exempt from Institutional Review Board approval.

### Study population

TriNetX was queried for patients undergoing robotic assisted pyeloplasty (RP; CPT 50544), ureteral reimplantation (RR; CPT 50947 and 50948), and ureteroplasty/ureteroureterostomy (RU; CPT 50949 and 50700). To specify a robotic assisted approach, the modifier code HCPCS (Healthcare Common Procedure Coding System) S2900 was used as well. The date of surgery was defined as the index event.

We created sub-queries to generate cohorts for risk factor analysis based on widely accepted risk factors for poor wound healing and surgical recovery. Specifically, for each of the three index procedures, we generated cohorts based on the presence or absence of vascular disease (ICD I25, I73.9, I70), smoking history (ICD F17.2, Z87.891, Logical Observation Identifiers Names and Codes 72166-2), chronic kidney disease (ICD N18), diabetes mellitus (ICD E8-13), radiation therapy (TriNetX code 1001, ICD Z92.3, CPT 1010843), body mass index (BMI) greater than or less than 25 kg/m^2^, and age greater than or less than 50.

### Outcomes and statistical analysis

Our primary outcome was need for a drain (percutaneous nephrostomy tube or ureteral stent; CPT 50040, 50432, 50694, 50695, 52332, 74480) 4 weeks to 10 years after index procedure. Many providers, including in our own practice, leave a stent in place at time of ureteral reconstruction, and this is often removed at some point 4–6 weeks postoperatively. For this reason, we felt that starting our time frame at 4 weeks would be an adequate time point. We choose drain placement as our primary outcome with the thinking that if a repair was to “fail” and obstruction to recur then at some point a patient would likely have a nephrostomy tube or stent placed to properly drain their kidney. There is no standard definition of success following upper tract repair, which is a limitation of the literature [9]. However, the need for subsequent procedures is a commonly used endpoint and this was the basis for our study. We acknowledge that some patients may have a drain placed for alternative reasons or patients may go on to have subsequent procedures without an interval drain placed and will expand upon this further in the limitations. We also acknowledge that this may miss other immediate postoperative complications such as anastomotic leak. With acknowledgement of the above, for the purpose of our study, ureteral stent or nephrostomy tube placement was the proxy for success we chose.

We evaluated drain placement following each of the index procedures. We also looked at drain placement for each combination of index procedure and risk factor. For the risk factor analysis, we performed propensity score matching (PSM) using the remaining risk factors as our covariates (i.e. for patients with diabetes vs. without diabetes, we used vascular disease, age, smoking history, radiation history, and BMI for PSM). TriNetX has developed their own platform so that users can perform PSM directly on their website by using 1:1 greedy nearest-neighbor PSM, a statistical technique that uses logistic regression to generate similarly sized cohorts based on variables of interest.

All analyses were performed internally via TriNetX on demographic data which calculated hazard ratios (HR) and 95% confidence intervals (CI). The 95%CIs that did not include 1.0 were considered significant. Final analyses were performed in October 2024. Of note, when less than 10 patients experience an outcome, TriNetX rounds the value to 10 to protect patient anonymity. Data points affected by this rounding mechanism are marked with an asterisk (*). P-values are reported when available based on patient volume in TriNetX.

## Results

We identified 1,324 patients who underwent RP, representing 30 different HCOs. There were 464 patients who underwent RR from 22 HCOs and 875 patients who underwent RU from 27 HCOs. Respectively, 10.8%, 11.7%, and 7.9% of patients needed a drain placed after 4 weeks postoperatively, resulting in success rates of 89.2%, 88.3%, and 92.1% (Table 1). The median follow-up and estimated 10-year survival for these procedures was 749 days and 82.8% for RP, 604 days and 81.7% for RR, and 377 days and 81.9% for RU.

**Table 1:** Drain placement following pyeloplasty, ureteral reimplant, and ureteroplasty

Procedure	Number	HCOs	Drain (nephrostomy or stent)
Pyeloplasty	1324	30	10.8%
Ureteral Reimplant	464	22	11.7%
Ureteroplasty	875	27	7.9%

HCO – Healthcare Organization, BMI – Body Mass Index (kg/m^2), PSM – Propensity Score Matching

Following RP, patients with a history of diabetes (18.8% vs. 10.4%, HR 2.0 (1.04–3.68)), smoking history (14.9% vs. 7.2%, HR 2.2 (1.34–3.49)), and BMI > 25 (12.7% vs. 7.6%, HR 1.6 (1.07–2.34)) showed a statistically significant increased risk of drain placement (Table 2). For the radiation sub-analysis, the overall number of patients undergoing pyeloplasty was low following PSM and our ability to interpret the impact of radiation is limited by the TriNetX rounding mechanism.

**Table 2:** Pyeloplasty risk factors and success

Risk factor	Number after PSM	Drain placement	Hazard ratio (95% Confidence Interval) *p*-value
+Diabetes -Diabetes	144	18.8% 10.4%	2.0 (1.04–3.68) *p* = 0.04
+Radiation -Radiation	20	< 50%* < 50%*	0.8 (0.17–4.15) Too few to report*
+Smoking -Smoking	349	14.9% 7.2%	2.2 (1.34–3.49) *p* < 0.01
+Vascular -Vascular	104	12.5% 17.3%	0.7 (0.35–1.46) *p* = 0.35
+Kidney -Kidney	151	10.6% 13.9%	0.7 (0.38–1.41) *p* = 0.23
Age > 50 years Age < 50 years	473	10.6% 11.2%	0.99 (0.68–1.46) *p* = 0.98
BMI > 25 kg/m^2 BMI < 25 kg/m^2	529	12.7% 7.6%	1.6 (1.07–2.34) *p* = 0.02

HCO – Healthcare Organization, BMI – Body Mass Index (kg/m^2), PSM – Propensity Score Matching

For RR, patients with a history of diabetes showed increased drain placement (23.5% vs. < 11.8*%, HR 2.8 (1.18–6.63)) (Table 3). This analysis is limited by the TriNetX rounding mechanism but if anything would show an increased difference since patients without diabetes had < 11.8% chance of having a drain placed. Radiation history also had a significantly increased association with drain placement (27.7% vs. < 21.3*%, HR 2.9 (1.03–8.09)), but the patients without a history of radiation were affected by TriNetX rounding. Age and BMI were additional risk factors with overall low numbers of drain placement and thus were impacted by the rounding.

**Table 3:** Ureteral reimplant risk factors and success

Risk factor	Number after PSM	Drain placement	Hazard ratio (95% Confidence Interval) *p*-value
+Diabetes -Diabetes	85	23.5% < 11.8%*	2.8 (1.18–6.63) Too few to report*
+Radiation -Radiation	47	27.7% < 21.3%*	2.9 (1.03–8.09) Too few to report*
+Smoking -Smoking	109	13.8% 12.8%	1.1 (0.52–2.22) *p* = 0.85
+Vascular -Vascular	80	18.8% 16.3%	1.3 (0.62–2.75) *p* = 0.48
+Kidney -Kidney	83	20.5% 16.9%	1.3 (0.66–2.73) *p* = 0.42
Age > 50 years Age < 50 years	147	< 6.8%* 8.8%	0.84 (0.37–1.91) Too few to report*
BMI > 25 kg/m^2 BMI < 25 kg/m^2	100	< 10%* < 10%*	0.73 (0.29–1.85) Too few to report*

HCO – Healthcare Organization, BMI – Body Mass Index (kg/m^2), PSM – Propensity Score Matching

For our final risk factor sub-analyses, following RU, patients with a radiation history (< 16.7*% vs. < 16.7*%, HR 3.7 (1.006–13.9)) and age > 50 years (9.5% vs. 3.9%, HR 2.3 (1.16–4.49)) showed increased association with drain placement, although the radiation sub-analysis is again heavily impacted by the TriNetX rounding (Table 4).

**Table 4:** Ureteroplasty risk factors

Risk factor	Number after PSM	Drain placement	Hazard ratio (95% Confidence Interval) *p*-value
+Diabetes -Diabetes	149	10.1% < 6.7%*	1.7 (0.75–3.93) Too few to report*
+Radiation -Radiation	60	< 16.7%* < 16.7%*	3.7 (1.006–13.9) Too few to report*
+Smoking -Smoking	237	10.1% 7.2%	1.4 (0.76–2.65) *p* = 0.26
+Vascular -Vascular	105	15.2% 10.5%	1.5 (0.69–3.25) *p* = 0.30
+Kidney -Kidney	124	16.1% 12.1%	1.3 (0.65–2.48) *p* = 0.49
Age > 50 years Age < 50 years	306	9.5% 3.9%	2.3 (1.16–4.49) *p* = 0.01
BMI > 25 kg/m^2 BMI < 25 kg/m^2	251	8.0% 8.0%	0.82 (0.44–1.52) *p* = 0.53

HCO – Healthcare Organization, BMI – Body Mass Index (kg/m^2), PSM – Propensity Score Matching

## Discussion

Our study represents the largest series to date regarding robotic upper urinary tract reconstruction, totaling over 2500 patients. Amongst all patients and procedures, the overall success rate was near 90%, which is comparable to previous literature [10–12]. Despite this, some of our analyses were still limited by overall sample size, particularly when assessing the risk for drain placement associated with a history of radiation. Diabetes was a risk factor with a significant association for both RP and RR. Age was an additional risk factor for RU and BMI and smoking history were additional risk factors for RP.

One of the weaknesses of the available literature on robotic upper urinary tract reconstruction comes from the strengths of the surgeons, particularly in regards to work on the ureter itself. Many of the studies on the topic come from groups such as CORRUS (the Collaborative of Reconstructive Robotic Ureteral Surgery) and former fellows that have trained under these surgeons. It is fair to question whether the surgical outcomes from these expert surgeons can be broadly applied and used to counsel patients undergoing procedures by other providers and at other institutions. From this collaborative, success rate following primary RP was 92%, their ureteroplasty success using a buccal graft was 89% while ureteroureterostomy was at 75%, and for distal strictures their success was over 90% regardless of technique, except for appendiceal bypass which was at 60%.^10–12^ Our study does not go into the same level of granularity on approach or stricture characteristics, but our outcomes are in a comparable range. We hope our data can provide further context for surgeons outside of these highly regarded centers when they counsel patients on what they can expect for success following their procedures.

There is relatively more data available on outcomes following RP [6], which both in our study and personal experience is a more commonly performed procedure. Pyeloplasty has demonstrated durable success with open, laparoscopic, and robotic approaches [13, 14]. The robotic assisted approach has gained favor as the new standard of care given its shorter learning curve and ease of suturing as well as some of the technological benefits mentioned previously [6, 15]. This especially holds true in cases of more challenging cases, such as a secondary/redo UPJ obstruction where the robotic approach has demonstrated comparable outcomes to primary pyeloplasty [10, 15]. 

Our findings are strengthened by the large sample size, long term follow up, as well as the use of PSM. With the utilization of the TriNetX database, we were able to generate a sample size larger than those previously reported [6, 15]. Additionally, we included follow-up up to 10 years, but do acknowledge our median follow-up is mostly comparable to other work [6, 15]. Previous studies looked at risk factors for surgical failure and found diabetes and obesity as notable factors for proximal/middle ureteral strictures and distal strictures respectively [12, 16]. Similarly in our work, diabetes and BMI were found to be risk factors for failure, in addition to age and smoking history. We did not look at ureteral rest (removing a ureteral stent prior to operation) as it would not be feasible to determine within TriNetX but this is a commonly evaluated factor for success [17]. We also did not evaluate race/ethnicity with stricture recurrence, which previous groups did not find to have a significant impact [18]. By using PSM, we felt that our study limits confounding to the best of our ability given the circumstances of using a large, anonymous, retrospective database.

With that said, our study is not without limitations. Our results are inherently subject to the limitations of all registry based, retrospective studies. We are dependent on the accuracy of the medical coders with inputting their data. Also, we lack granular detail and have no ability to examine individual patient charts. Therefore, we had no ability to determine important factors such as stricture length, location, multifocality, etc. We also did not investigate whether there was any impact on success if patients received adjunct procedures, such as a boari flap or buccal graft, but this could be a topic for future study. This study also did not focus on the distinction between primary and redo operations which are known to be more challenging. We decided to choose drain placement as a marker for surgical success but recognize there is no widely established definition of success which limits our analyses. Further, some patients may require drain placement for alternative reasons and not just surgical failure. However, if this were to be the case, it would only suggest that the success rates are even higher. Of course, patients may also present outside of a TriNetX institution in which case some failures may be underreported. When using registry data, tradeoffs must be made between inclusivity and specificity to generate cohorts, wherein we prioritized inclusivity. Future studies could prioritize more specific clinical scenarios and comparisons, but this was outside the scope of our current work. We also were unable to directly evaluate factors such as surgeon or HCO volume, and although a strength of our study was the ability to evaluate a large number of diverse practices, we cannot report on quite how high or low volume the HCOs we evaluated actually are. It certainly would be interesting to perform a study comparing surgical experience, surgeon skill as judged by video analysis, and reconstructive success, but that is currently outside the scope of this study.

This study presents the largest series to date using real world data regarding the general success rate of various upper urinary tract reconstruction procedures from many different HCOs. We also present data on risk factors that were associated with decreased surgical success. The sheer number of patients coming from many different HCOs is novel data amongst this field. We feel our data will be of benefit to surgeons when counseling their patients and help properly set expectations. Of note, we did not attempt to exclude higher volume surgeons. Instead we intended to include a larger volume of surgeons to generate more diverse surgical expertise and experience, which we feel supports the generalizability of our results. Future work can be directed towards specific clinical scenarios, such as the use of grafts or flaps and comparing primary vs. redo procedures, and interested surgeons should still exercise substantial caution when considering more complex repairs.

## Conclusions

Robotic upper urinary tract reconstruction is a growing field but with most of the data coming from a few high-volume expert surgeons. We provide real world data to help surgeons properly counsel their patients regarding surgical success from a diverse collection of HCOs. Additionally, various potential risk factors for surgical failure were assessed following propensity score matching to determine their association with surgical failure. The authors have no funding or conflicts of interest to report.

## Data Availability

No datasets were generated or analysed during the current study.
